# Supplementary material for: Mapping the evolution of fertility support policies in China: A content and instrumental analysis
Source: PLoS One. 2025 Oct 9;20(10):e0332137. doi: 10.1371/journal.pone.0332137 (PMC12510515; doi:10.1371/journal.pone.0332137)
Supplement: S1 Appendix — (ZIP) [file pone.0332137.s001.zip › S1 Appendix. 226 original policy documents/18-国家卫生和计划生育委员会关于全面开展创建幸福家庭活动的通知(FBM-CLI-4-226981).doc]

国家卫生和计划生育委员会关于全面开展创建幸福家庭活动的通知

**发布部门：** [国家卫生和计划生育委员会(已撤销)](https://www.pkulaw.com/law/chl?Aggs.IssueDepartment=60319) [机构沿革](javascript:void(0))

**发文字号：**国卫家庭发〔2014〕28号

**发布日期：**2014.05.16

**实施日期：**2014.05.16

**时效性：** [现行有效](https://www.pkulaw.com/law/chl?Aggs.TimelinessDic=01)

**效力级别：** [部门工作文件](https://www.pkulaw.com/law/chl?Aggs.EffectivenessDic=XE0303)

**法规类别：** [家庭关系](https://www.pkulaw.com/law/chl?Aggs.Category=02204)

国家卫生和计划生育委员会关于全面开展创建幸福家庭活动的通知
（国卫家庭发[2014]28号）

各省、自治区、直辖市卫生计生委（人口计生委），计划单列市及新疆生产建设兵团卫生计生委（人口计生委），解放军、武警部队计划生育领导小组办公室，中直机关、中央国家机关人口计生委：
　　2011年，原国家人口计生委、中国计划生育协会、中国人口福利基金会启动创建幸福家庭活动试点工作。2013年国家卫生计生委组建以来，继续深入推进创建幸福家庭活动试点工作。3年来，全国各地特别是12个试点省份和140个试点市紧紧围绕“文明、健康、优生、致富、奉献”主题，扎实开展“宣传倡导、健康促进、致富发展”三大活动，为城乡家庭特别是计划生育家庭做好事、办实事、解难事，促进了计生工作转型发展，密切了党群干群关系，增进了社会和谐。创建活动已经成为提升家庭幸福感的切实举措，成为百姓普遍认可的知名品牌。试点工作取得的积极成效，为在全国范围广泛开展创建幸福家庭活动奠定了坚实基础。为深入贯彻党的十八大和十八届三中全会精神，进一步做好卫生计生工作，促进家庭和谐幸福，国家卫生计生委决定在全国全面开展创建幸福家庭活动。现就有关事项通知如下：
　　**一、**重大意义
　　（一）创建幸福家庭活动，是实现中国梦的重要途径。中华民族伟大复兴的中国梦是中华儿女的共同期盼，其出发点和落脚点都是为人民谋幸福。卫生计生工作的重要任务，就是不断提高人民健康水平，促进人的全面发展、家庭幸福和社会和谐。创建幸福家庭活动，就是要通过守护好健康这个幸福的起点，落实好计划生育基本国策，创造实现人民幸福的良好人口环境，顺应和满足人民群众对美好生活的新期待。
　　（二）创建幸福家庭活动，是贯彻社会主义核心价值观的具体实践。社会主义核心价值观的重要内容，与每个人和每个家庭息息相关。创建幸福家庭活动，就是要通过弘扬“尊老爱幼、男女平等、勤俭持家”等传统美德，倡导“以家庭和睦促进环境和美、邻里和气、社区和善、社会和谐”，生动全面地诠释和践行社会主义核心价值观。
　　（三）创建幸福家庭活动，是改进社会治理方式的有效载体。社会领域改革具有系统性和协同性，根本目的是增进人民福祉。创建幸福家庭活动就是要通过转变政府职能，鼓励和动员全社会力量完善家庭发展政策，提高家庭发展能力，围绕充分就业、教育公平、社会保障、病有所医和老有所养等内容，建立健全党政统筹、社会协同、公众参与的社会工作格局，从而提升社会治理的能力和水平。

　　**二、**总体目的和主要任务
　　（一）总体目的。围绕“文明、健康、优生、致富、奉献”主题，弘扬中华民族传统美德，推动社会主义核心价值观及文明理念更加深入人心；提高全民健康、计划生育和生殖健康水平，促进出生人口性别平衡；健全完善有利于家庭发展的经济社会政策，全面提高家庭发展能力、生活质量和幸福指数。
　　（二）主要任务。
　　1.加强宣传倡导，促进家庭文明。注重人口文化及家庭发展理论研究，大力弘扬中华民族尊老爱幼、男女平等、邻里互助等传统美德，以“富强、民主、文明、和谐”的国家政治理想、“自由、平等、公正、法治”的社会价值取向与“爱国、敬业、诚信、友善”的个人道德准则，引领良好家风家规，培养家庭成员文明健康、低碳环保、友爱奉献的生活习惯与方式，做到文明立家、健康安家、优生乐家、致富兴家、奉献传家。利用群众喜闻乐见的多种宣传形式，营造落实计划生育基本国策的良好氛围，引导群众依法生育，澄清社会上对计划生育工作的模糊和错误认识，打造家庭建设品牌，传播家庭幸福正能量。
　　2.加强公共服务，守护家庭健康。整合加强人口信息平台建设，及时掌握人口基础信息和群众卫生计生服务需求。大力开展健康促进行动，广泛传播健康科学知识和家庭文明理念，帮助群众树立健康意识，培养形成健康文明的生活方式。发挥行业优势，以需求为导向，针对全人口和生命全过程，立足家庭提供计划生育、优生优育、生殖健康、不孕不育诊治、青少年健康发展、老年健康、助老养老等系列公共服务。基层卫生、妇幼保健和计划生育技术服务机构积极主动、全面开展免费孕前优生健康检查、“两癌”筛查和生殖道感染普查普治等基本预防保健和医疗卫生服务。在切实维护群众身体健康的同时，帮助保持积极健康心态，及时疏缓心理压力，构建和谐人际关系，促进家庭成员的全面发展。健全完善工作机制，加大人员、资金、设备和技术投入保障力度。
　　3.加强资源整合，帮扶家庭致富。针对家庭在生产、生活、生育等方面存在的不同困难和问题，协调动员社会资源切实开展扶贫济困、致富发展工作。整合扶贫项目资金，开展医疗救助，减少因病致贫、因病返贫；组织生产经营项目、资金和技术进街道社区，进村入户，积极构建“扶持、优惠、保障、奖励、资助、关怀”政策体系，提供个性化、多样化的脱贫致富支持；支持城乡家庭特别是计划生育困难家庭发展生产、勤劳致富，实现收入增加、生活宽裕、富足祥和、安居乐业。 进一步完善计划生育家庭奖励扶助和特别扶助制度，着力帮助解决计划生育特殊困难家庭的现实问题。重视计划生育家庭养老问题，优先纳入社会养老服务体系。对失能老人家庭、残疾人家庭、流动人口家庭和留守家庭等，给予针对性的扶助关爱，努力让所有家庭共同发展。
　　4.强化社会责任，引领家庭奉献。通过强化家庭的自我教育、自我要求，培养家庭的责任意识、感恩情怀和回报愿望，正确对待小家与大家、个人与社会的关系，实现家庭道德修养的自我升华。鼓励率先过上富裕幸福生活的家庭，以先富带动后富，以先发展带动后发展，带动大家走共同富裕的幸福之路。组织开展形式多样的幸福家庭创先争优活动，树立推出典型模范，宣传学习先进事迹，让幸福家庭的奉献付出影响带动邻里、社区以至全社会。努力让每个家庭成为扶弱济困的慷慨解囊者，见义勇为的正义维护者，热心公益事业的志愿者，构建和谐社会的建设者，通过爱心温暖他人，通过行动奉献社会。通过开展创建活动，引领“我为人人、人人为我”的社会道德风尚，努力实现个人为家庭奉献、家庭为社会奉献，全社会关爱每个家庭、每个家庭得到全面发展的良性循环。

　　**三、**有关要求
　　（一）提高认识，强化领导。健全完善党委政府领导统筹、卫生计生部门牵头组织、相关部门配合联动、城乡群众广泛参与的长效工作机制，把创建活动纳入本地经济社会发展规划和改善民生的重要内容，确保领导到位、责任到位、投入到位、工作到位。
　　（二）依托项目，拓展内容。以创建幸福家庭活动为综合性载体，设计项目，整合资源，引导各级财政资金、社会资源向幸福家庭建设上倾斜。实施拓展“新家庭计划”、全民健康素养促进活动、免费孕前优生健康检查、“少生快富”工程、幸福工程、生育关怀等品牌项目。充分发挥社会组织的作用，鼓励承接政府购买服务，提供贴近群众需求的家庭服务产品。
　　（三）目标管理，示范引领。将创建幸福家庭活动纳入计划生育目标管理责任制，通过绩效考核、检查评估等方式，促进各项工作任务落实。鼓励地方研究制订符合当地实际和活动特点的创建标准、评估内容和考核方法，引导并规范本地创建工作。培养推出一批创建幸福家庭活动示范市，发挥示范带动作用，推动基层探索创新，营造创先争优的良好氛围，推动创建幸福家庭活动持续健康发展。

国家卫生计生委
2014年5月16日

© 北大法宝：（[www.pkulaw.com](https://www.pkulaw.com/)) 专业提供法律信息、法学知识和法律软件领域各类解决方案。北大法宝为您提供丰富的参考资料，正式引用法规条文时请与标准文本核对。
欢迎查看所有[产品和服务](https://www.pkulaw.com/helps/2.html)。法宝快讯：[如何快速找到您需要的检索结果？](https://www.pkulaw.com/helps/70.html)[法宝](https://www.pkulaw.com/helps/69.html)V6有何新特色？

扫描二维码阅读原文

**原文链接：**<https://www.pkulaw.com/chl/a87d381fd93e8176bdfb.html>
